# Community transcriptomics reveals universal patterns of protein sequence conservation in natural microbial communities

**DOI:** 10.1186/gb-2011-12-3-r26

**Published:** 2011-03-22

**Authors:** Frank J Stewart, Adrian K Sharma, Jessica A Bryant, John M Eppley, Edward F DeLong

**Affiliations:** 1 School of Biology, Georgia Institute of Technology, Ford ES&T Building, Rm 1242, 311 Ferst Drive, Atlanta, GA 30332, USA; 2 Department of Civil and Environmental Engineering, Massachusetts Institute of Technology, Parsons Laboratory 48, 15 Vassar Street, Cambridge, MA 02139, USA

## Abstract

**Background:**

Combined metagenomic and metatranscriptomic datasets make it possible to study the molecular evolution of diverse microbial species recovered from their native habitats. The link between gene expression level and sequence conservation was examined using shotgun pyrosequencing of microbial community DNA and RNA from diverse marine environments, and from forest soil.

**Results:**

Across all samples, expressed genes with transcripts in the RNA sample were significantly more conserved than non-expressed gene sets relative to best matches in reference databases. This discrepancy, observed for many diverse individual genomes and across entire communities, coincided with a shift in amino acid usage between these gene fractions. Expressed genes trended toward GC-enriched amino acids, consistent with a hypothesis of higher levels of functional constraint in this gene pool. Highly expressed genes were significantly more likely to fall within an orthologous gene set shared between closely related taxa (core genes). However, non-core genes, when expressed above the level of detection, were, on average, significantly more highly expressed than core genes based on transcript abundance normalized to gene abundance. Finally, expressed genes showed broad similarities in function across samples, being relatively enriched in genes of energy metabolism and underrepresented by genes of cell growth.

**Conclusions:**

These patterns support the hypothesis, predicated on studies of model organisms, that gene expression level is a primary correlate of evolutionary rate across diverse microbial taxa from natural environments. Despite their complexity, meta-omic datasets can reveal broad evolutionary patterns across taxonomically, functionally, and environmentally diverse communities.

## Background

Variation in the rate and pattern of amino acid substitution is a fundamental property of protein evolution. Understanding this variation is intrinsic to core topics in evolutionary analysis, including phylogenetic reconstruction, quantification of selection pressure, and identification of proteins critical to cellular function [1,2]. A diverse range of factors has been postulated to affect the rate of sequence evolution within individual genomes, including mutation and recombination rate [3], genetic contributions to fitness (that is, gene essentiality) [4], timing of replication [5], number of protein-protein interactions [6-8], and gene expression level [9]. Among these, gene expression level has emerged as the strongest predictor of evolutionary rate across diverse taxa, with highly expressed genes experiencing high sequence conservation [9-14]. However, these studies have focused on model organisms or small numbers of target species. The links between gene expression and broader evolutionary properties, including evolutionary rate, and the mechanistic basis for these relationships remain poorly described for the vast majority of organisms, notably non-model taxa from diverse natural communities.

Deep-coverage sequencing of microbial community DNA and RNA (metagenomes and metatranscriptomes) provides an unprecedented opportunity to explore protein-coding genes across diverse organisms from natural populations. Such studies have yielded valuable insight into the genetic potential and functional activity of natural communities [15-19], but thus far have been applied only sparingly to questions of evolution. Furthermore, only a subset of studies present coupled DNA-RNA datasets for comparison [17,19-21]. When analyzed in tandem, coupled DNA-RNA datasets facilitate categorization of the relative transcription levels of different gene categories, potentially revealing properties of sequence evolution driven in part by expression level variation. However, it remains uncertain whether broad evolutionary correlates of gene expression, potentially including sequence conservation, would even be detectable in community-level samples, which contain sequences from potentially thousands of widely divergent taxa. Here, we compare microbial metagenomic and metatranscriptomic datasets from marine and terrestrial habitats to explore fundamental properties of sequence evolution in the expressed gene set.

Specifically, we use coupled microbial (Bacteria and Archaea) metagenomic and metatranscriptomic datasets to explore the hypothesis that highly expressed genes are more conserved than minimally expressed genes. In lieu of conservation estimates based on alignments of orthologous genes, which are not feasible using fragmentary shotgun data containing tens of thousands of genes, sequence conservation was estimated based on amino acid identity relative to top matches in a reference database. Our results indicate a strong inverse relationship between evolutionary rate and gene expression level in natural microbial communities, measured here by proxy using transcript abundance. Furthermore, these results demonstrate broad consistencies in protein-coding gene expression, amino acid usage, and metabolic function across ecologically and taxonomically diverse microorganisms from different environments. This study illustrates the utility of environmental meta-omic datasets for informing theoretical predictions based (largely) on model organisms in controlled laboratory settings.

## Results and discussion

### Expressed genes evolve slowly

The relationship between gene expression (transcript abundance) and sequence conservation was examined for protein-coding genes in coupled metagenome and metatranscriptome datasets generated by shotgun pyrosequencing of microbial community DNA and RNA, respectively. These datasets represent varied environments, including the oligotrophic water column from two subtropical open ocean sites in the Bermuda Atlantic Time Series (BATS) and Hawaii Ocean Time Series (HOT) projects, the oxygen minimum zone (OMZ) formed in the nutrient-rich coastal upwelling zone off northern Chile, and the surface soil layer from a North American temperate forest (Tables 1 and 2). Prior studies have experimentally validated the metatranscriptomic protocols used here (RNA amplification, cDNA synthesis, pyrosequencing; see Materials and methods), confirming that estimates of relative transcript abundance inferred from pyrosequencing accurately parallel measurements based on quantitative PCR [15,17,19]. Here, amino acid identity relative to a top match reference sequence identified by BLASTX against the National Center for Biotechnology Information non-redundant protein database (NCBI-nr) is used to estimate sequence conservation.

**Table 1:** Read counts and accession numbers of pyrosequencing datasets

			**Sequences**^**a**^			
				
Site	Depth (m)	Data	Total	**Non-rRNA**^**b**^	**Coding**^**c**^	Accession
OMZ	50	DNA	393,403	340,117	204,953	SRX025906
		RNA	379,333	117,760	42,327	SRX025907
	85	DNA	595,662	567,772	341,350	SRX025908
		RNA	184,386	69,200	16,960	SRX025909
	110	DNA	403,227	380,057	215,217	SRX025910
		RNA	557,762	268,093	81,492	SRX025911
	200	DNA	516,426	485,044	274,463	SRX025912
		RNA	441,273	149,699	39,218	SRX025913
BATS 216	20	DNA	357,882	343,370	223,563	SRX008032
		RNA	511,525	334,507	124,832	SRX016882
	50	DNA	464,652	423,258	244,638	SRX008033
		RNA	365,838	263,811	91,489	SRX016883
	100	DNA	525,606	498,222	305,260	SRX008035
		RNA	519,951	334,037	129,369	SRX016884
HOT 186	25	DNA	623,559	596,902	331,347	SRX007372
		RNA	561,821	252,586	113,664	SRX016893
	75	DNA	995,747	654,106	363,459	SRX007369
		RNA	557,718	199,416	55,545	SRX016897, SRX016896
	110	DNA	473,166	458,260	237,759	SRX007370
		RNA	398,436	135,452	34,644	This study, SRA028811
	500	DNA	673,674	972,967	540,042	SRX007371
		RNA	479,661	83,795	38,913	This study, sra028811
Soil	Surface	DNA	1,439,445	1,392,745	976,899	This study, sra028811
		RNA	1,188,352	985,305	445,479	This study, sra028811

^a^Generated on a Roche 454 GS FLX instrument. ^b^All non-rRNA reads; duplicate reads (reads sharing 100% nucleotide identity and length) excluded. ^c^Reads matching (bit score >50) protein-coding genes in the NCBI-nr database. BATS, Bermuda Atlantic Time Series; HOT, Hawaii Ocean Time Series; OMZ, oxygen minimum zone; rRNA, ribosomal RNA.

**Figure 1:**
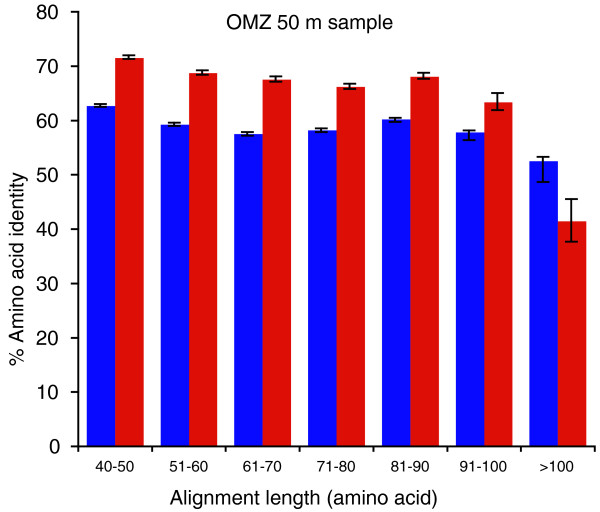
**Discrepancies in DNA (blue) and RNA (red) amino acid identities over variable high-scoring segment pair alignment lengths**. Reads were binned by HSP alignment length, with identities averaged across all genes identified per bin. Error bars are 95% confidence intervals.

In all the samples, amino acid identities, averaged across all genes per dataset, were significantly higher for RNA-derived sequences (metatranscriptomes) compared to DNA-derived sequences (metagenomes), with an average difference of 8.9% between paired datasets (range, 4.4 to 14.7%; *P *< 0.001, *t*-test; Table 2). Further analysis of a representative sample (OMZ, 50 m) showed that RNA identities remained consistently elevated across a gradient of high-scoring segment pair (HSP) alignment lengths (Figure 1). This pattern suggests that the DNA-RNA difference was not driven by the (on average) shorter read lengths in the RNA transcript pool (length data not shown), which could have imposed selection for reads with higher identity in order to meet the bit score cutoff (see Materials and methods). This pattern was not observed in the highest alignment length bin (>100 amino acids), likely due to the small number of genes (*n *= 53) detected among the RNA reads falling into this category (for example, 0.4% of those in the 40 to 50 amino acid bin; see error bars in Figure 1).

**Table 2:** Mean percentage amino acid identity of 454 reads matching database reference genes (NCBI-nr) shared between and unique to DNA and RNA samples

			**Percentage identity to reference genes present in**^**a**^			
			
	Depth (m)	Data	**DNA+RNA**^**b**^	**DNA only**^**c**^	RNA only	**All**^**d**^
OMZ	50	DNA	71.0	59.8	NA	60.8
		RNA	73.8	NA	72.2	72.7
	85	DNA	67.3	59.5	NA	59.8
		RNA	68.4	NA	67.9	68.1
	110	DNA	65.7	58.7	NA	59.7
		RNA	68.5	NA	71.1	70.2
	200	DNA	64.3	58.5	NA	59.1
		RNA	67.0	NA	65.9	66.4
BATS 216	20	DNA	72.5	59.5	NA	62.7
		RNA	75.6	NA	71.6	72.9
	50	DNA	76.4	61.5	NA	64.4
		RNA	78.3	NA	71.2	74.1
	100	DNA	76.8	60.5	NA	63.9
		RNA	78.6	NA	71.6	74.8
HOT 186	25	DNA	75.3	63.7	NA	65.7
		RNA	76.4	NA	69.1	72.0
	75	DNA	77.3	64.1	NA	65.6
		RNA	77.5	NA	69.1	72.9
	110	DNA	80.0	60.7	NA	62.4
		RNA	81.3	NA	73.0	77.1
	500	DNA	63.1	59.4	NA	59.6
		RNA	64.0	NA	66.0	65.0
Soil	Surface	DNA	58.9	55.0	NA	56.1
		RNA	59.8	NA	61.1	60.5

^a^Mean percentage identity across all genes (unique accession numbers) identified via BLASTX against NCBI-nr (HSP alignment regions only; bit score cutoff = 50). ^b^Genes present in both DNA and RNA datasets, that is, 'expressed' genes. ^c^Genes present only in the DNA dataset, that is, 'non-expressed' genes. ^d^Genes shared between datasets (in DNA + RNA) plus genes unique to a dataset. BATS, Bermuda Atlantic Time Series; BLAST, Basic Local Alignment Search Tool; HOT, Hawaii Ocean Time Series; HSP, high-scoring segment pair; NA, not applicable; NCBI-nr, National Center for Biotechnology Information non-redundant protein database; OMZ, oxygen minimum zone; rRNA, ribosomal RNA.

To further rule out that the DNA-RNA discrepancy was due to methodological differences in DNA- and RNA-derived samples (for example, error rate variation due to differential sample processing; see Materials and methods), we examined amino acid identities in expressed and non-expressed genes derived from the DNA dataset only. Hereafter, we operationally define 'non-expressed' genes as those detected only in the DNA datasets, whereas 'expressed' genes are those detected in both the DNA and RNA datasets (gene counts per fraction are provided in Table 3). Across all datasets, mean identities for DNA-derived non-expressed genes were significantly lower (mean difference, 10.6%; range, 3.7 to 19.4%; *P *< 0.001, *t*-test; Table 2) than those of DNA-derived expressed genes, whose values were similar to those of RNA transcripts that matched expressed genes (Table 2). This trend was consistent across all samples (Table 2) and independent of the database used for identifying reads, as comparisons against the Kyoto Encyclopedia of Genes and Genomes (KEGG) and Global Ocean Sampling (GOS) protein databases for a representative sample (OMZ, 50 m) revealed a similar RNA-DNA incongruity (Table 4). Furthermore, this pattern was unchanged when ribosomal proteins were excluded from the datasets (Table 4), as has been done previously to avoid bias due to the high expression and conservation of these proteins [14]. These data confirm a significantly higher level of sequence conservation in expressed versus non-expressed genes, broadly defined based on the presence or absence of transcripts.

**Table 3:** Unique reference genes shared between and unique to DNA and RNA datasets

		**Reference genes present in**^**a**^		
		
	Depth (m)	**DNA+RNA**^**b**^	**DNA only**^**c**^	RNA only
OMZ	50	11,374	113,747	21,445
	85	5801	172,055	6766
	110	17,843	109,924	31,697
	200	12,688	126,574	17,408
BATS 216	20	29,841	90,866	60,287
	50	26,954	110,131	38,145
	100	31,416	119,795	36,871
HOT 186	25	28,459	135,390	44,243
	75	18,098	142,892	21,800
	110	12,148	125,882	12,315
	500	14,345	248,534	13,573
Soil	Surface	104,453	283,180	107,475

^a^Number of unique NCBI-nr reference genes (accession numbers) identified as top matches to query reads via BLASTX (bit score > 50); in instances when a read matched multiple genes with equal bit scores, all genes were counted. ^b^Genes present in both DNA and RNA datasets, that is, 'expressed' genes. ^c^Genes present only in the DNA dataset, that is, 'non-expressed' genes. BATS, Bermuda Atlantic Time Series; BLAST, Basic Local Alignment Search Tool; HOT, Hawaii Ocean Time Series; NCBI-nr, National Center for Biotechnology Information non-redundant protein database; OMZ, oxygen minimum zone.

**Table 4:** Mean percentage amino acid identity of OMZ 50-m reads with top matches to distinct reference databases (GOS, KEGG, NCBI-nr) and with ribosomal proteins removed

			**Percentage identity to reference genes present in**^**b**^			
			
	**Database**^**a**^	Data	**DNA+RNA**^**c**^	**DNA only**^**d**^	RNA only	**All**^**e**^
All data						
	GOS	DNA	89.3	82.1	NA	82.8
	GOS	RNA	90.8	NA	87.5	89.3
	KEGG	DNA	67.8	58.3	NA	59.7
	KEGG	RNA	71.0	NA	69.4	69.6
	NR	DNA	71.0	59.8	NA	60.8
	NR	RNA	73.8	NA	72.2	72.7
Without ribosomal proteins^f^						
	NR	DNA	70.7	59.6	NA	60.6
	NR	RNA	73.6	NA	71.9	72.5

^a^BLAST database against which reads were compared. ^b^Mean percentage identity across all genes identified via BLASTX against NCBI-nr (HSP alignment regions only; bit score cutoff = 50). ^c^Genes present in both DNA and RNA datasets, that is, 'expressed' genes. ^d^Genes present only in the DNA dataset, that is, 'non-expressed' genes. ^e^Genes shared between datasets (in DNA + RNA) plus genes unique to a dataset. ^f^Ribosome-associated proteins removed manually from datasets. BATS, Bermuda Atlantic Time Series; BLAST, Basic Local Alignment Search Tool; GOS, Global Ocean Sampling; HOT, Hawaii Ocean Time Series; HSP, high-scoring segment pair; KEGG, Kyoto Encyclopedia of Genes and Genomes; NA, not applicable; NR, National Center for Biotechnology Information non-redundant protein database (NCBI-nr); OMZ, oxygen minimum zone.

**Figure 2:**
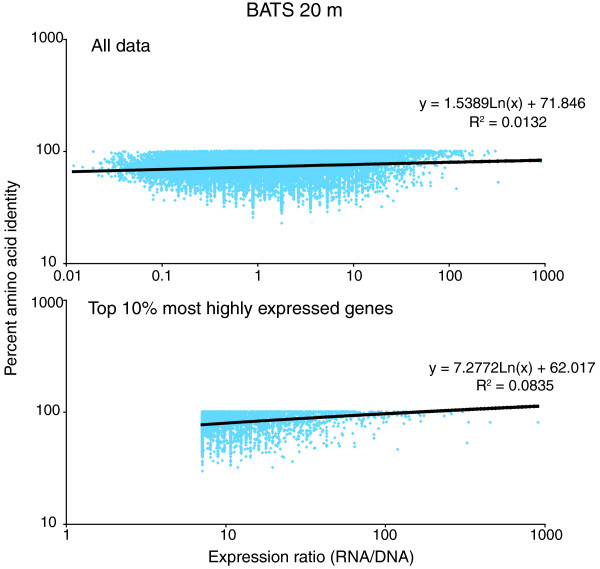
**Percentage amino acid identity as a function of expression level in the Bermuda Atlantic Time Series 20 m sample**. Per gene expression level is measured as a ratio - (Transcript abundance in RNA sample)/(Gene abundance in the DNA sample) - with abundance normalized to dataset size. Per gene percentage amino acid identity is averaged over all reads with top BLASTX matches to that gene.

**Figure 3:**
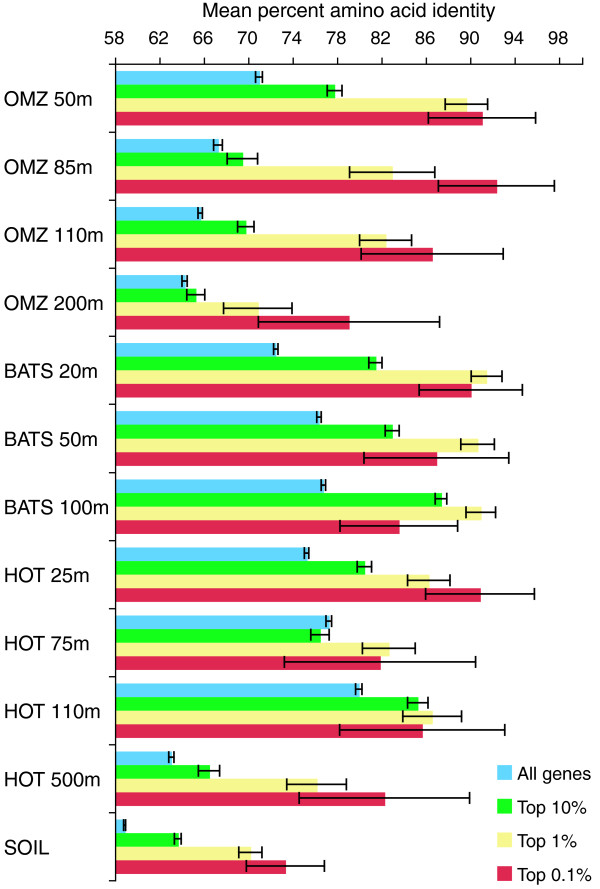
**Sequence conservation increases with mRNA expression ratio**. Genes are binned by rank expression ratio: all genes, top 10%, 1%, and 0.1% most highly expressed. Amino acid sequence identity is averaged across all DNA reads per gene (HSP alignment regions only), and then across all genes per bin. Error bars are 95% confidence intervals.

Given the differences observed between expressed and non-expressed categories, a positive correlation between conservation and the relative level of gene expression may also be anticipated [9]. Here, per-gene expression level was measured as the ratio of gene transcript abundance in the RNA relative to gene abundance in the DNA, with abundance normalized to dataset size. Correlations between amino acid identity and expression ratio were not observed in any of the samples when all genes representing all taxa were combined (r^2 ^= 0 to 0.02; see Figure 2 for a representative dataset). This pattern suggests that for a substantial portion of the metatranscriptome, transcriptional activity cannot be used as a predictor of evolutionary rate. This is likely due in part to the difficulty of accurately estimating expression ratios for low frequency genes, which constitute the majority of the metatranscriptome at the sequencing depths used in this study [22,23]. However, across all samples, mean amino acid identity consistently increased with expression ratio when genes were binned into broad categories: all genes, top 10%, top 1%, and top 0.1% most highly expressed (Figure 3). These data indicate that while transcript abundance is a poor quantitative indicator of sequence conservation on a gene-by-gene basis in community datasets, the most highly expressed genes are, on average, more highly conserved than those expressed at lower levels.

### Genome-level corroboration

It is possible that differences in the relative representation of genes in the BLAST databases may cause the incongruity in sequence conservation between expressed and non-expressed genes. Specifically, if expressed genes are more abundant in the database (which may be likely if these genes are also more abundant in nature), an expressed gene sampled from the environment will have a higher likelihood of finding a close match in the database, relative to a non-expressed gene. We therefore examined the discrepancy between expressed and non-expressed gene sets only for DNA reads whose top hits match the same reference genome. Under a null hypothesis of uniform evolutionary rates across a genome, all genes in a sample whose closest relative is the same reference genome should exhibit uniform divergence from the reference.

**Figure 4:**
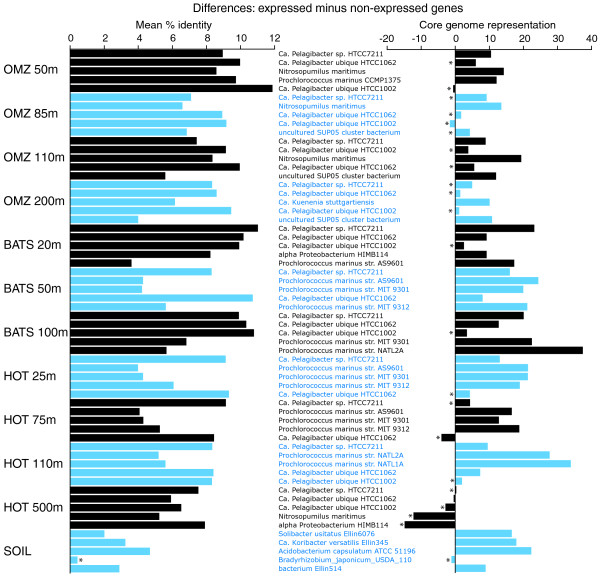
**Expressed and non-expressed genes differ in amino acid identity (left) and core genome representation (right)**. Data are from DNA sequence sets and include the five most abundant taxa per sample, with taxon abundance determined by the proportion of total reads with top matches to protein-coding genes in each genome (BLASTX of all DNA reads against NCBI-nr). 'Core genome representation' is calculated as the percentage of each gene set (that is, expressed or non-expressed genes) falling within the core genome of each taxon, as defined in the text. All differences (left and right panels) are significant (*P *< 0.001), unless marked with an asterisk.

The link between expression level and sequence conservation was observed at the level of individual genomes. Figure 4 (left panel) shows the discrepancy in amino acid identity between expressed versus non-expressed genes that match the top five most abundant reference taxa (whole genomes) in each sample. In all genomes, excluding *Bradyrhizobium japonicum *from the soil sample, the mean amino acid identity of expressed genes was significantly greater than that of non-expressed genes (*P *< 0.001, *t*-test). These taxon-specific patterns argue against an overall bias due to varying levels of gene representation in the database. Rather, assuming that the sequences that match the expressed and non-expressed gene fractions of a given reference genome are indeed present in the same genome in the sampled environment (an assumption that might be unwarranted if these two gene fractions experience varying rates of recombination or horizontal transfer among divergent taxa - see below), these results suggest that differential conservation levels, and not sampling artifacts, are driving the overall discrepancy between expressed and non-expressed genes.

### Core genes are overrepresented in the expressed gene fraction

Our data confirm an inverse relationship between expression level and evolutionary rate in natural microbial communities. However, it remains unclear to what extent gene expression level depends on a gene's functional importance to organism fitness (that is, essentiality) versus other potential explanations, such as 'translational accuracy or robustness' [24]. It has been argued that orthologous genes retained across divergent taxa ('core' genes) may mediate basic cellular functions and that such genes are more likely to be more essential than non-core (taxon-specific) genes [25-27]. Here, we calculated the proportional representation of expressed and non-expressed genes in the core genome, determined separately for each of the top five most abundant organisms in each of the samples (18 taxa total). Each taxon's core genome is composed of a relative orthologous gene set determined from comparison to a closely related sister taxon (or taxa; Table 5). The exact number of genes within each core set would likely vary if different sister taxa were used for comparison [28]. Here, the proportion of each genome that fell within the core set varied widely, from 17 to 80% (Table 5), reflecting natural variation and variation in the availability of whole genomes from different taxonomic groups.

**Table 5:** Proportion of reference taxon genes shared with sister taxon (that is, core gene set)

**Taxon**^**a**^	**Number of CDS**^**b**^	**Sister taxon**^**c**^	**Percentage of core**^**d**^
Alpha Proteobacterium *HIMB114*	1,425	*Pelagibacter ubique *HTCC1062	63
Ca. *Kuenenia stuttgartiensis*	4,787	*Planctomyces limnophilus *DSM 3776	17
*Nitrosopumilus maritimus*	1,796	*Cenarchaeum symbiosium*	49
Ca. *Pelagibacter *sp. HTCC7211	1,447	*Pelagibacter ubique *HTCC1062	75
Ca. *Pelagibacter ubique *HTCC1002	1,423	*Pelagibacter *sp. HTCC7211	80
Ca. *Pelagibacter ubique *HTCC1062	1,354	*Pelagibacter *sp. HTCC7211	80
*Prochlorococcus marinus *AS9601	1,920	All *Pro. *strains	68
*Prochlorococcus marinus *CCMP1375	1,883	All *Pro. *strains	69
*Prochlorococcus marinus *MIT 9312	1,810	All *Pro. *strains	72
*Prochlorococcus marinus *MIT9301	1,906	All *Pro. *strains	67
*Prochlorococcus marinus *NATL1A	2,193	All *Pro. *strains	59
*Prochlorococcus marinus *NATL2A	2,162	All *Pro. *strains	59
Uncultured *SUP05 *cluster bacterium	1,456	Ca. *Ruthia magnifica*	52
*Solibacter usitatus *Ellin6076	7,826	*Acidobacterium capsulatum *ATCC 51196	22
Ca. *Koribacter versatilis *Ellin345	4,777	*Acidobacterium capsulatum *ATCC 51196	36
*Acidobacterium capsulatum *ATCC 51196	3,377	*Solibacter usitatus *Ellin6076	51
*Bradyrhizobium japonicum *USDA 110	8,317	*Bradyrhizobium *sp. BTAi1	49
Bacterium Ellin514	6,510	*Verrucomicrobium spinosum *DSM 4136	24

^a^Representative taxon at high abundance in each sample. ^b^Number of CDS is the number of protein-coding genes in the sequenced reference genome of each taxon. ^c^Sister taxon used for identification of core genome (see main text). ^d^Percentage of core is the percentage of protein-coding genes in each taxon that are shared with the sister taxon. CDS, coding sequence.

Expressed genes were significantly more likely to fall within a core gene set shared across taxa. Figure 4 (right panel) shows the difference in core genome representation (percentage of genes within core set) between expressed and non-expressed gene fractions for each reference organism. In 52 of the 60 comparisons (87%), the percentage of expressed genes falling within the core set was greater than that for the non-expressed gene fraction; of these differences, 38 (73%) were significant (*P *< 0.0009, chi-square). In some taxa, such as *Prochlorococcus marinus *str. NATL2A, core genome representation was over 30% greater among expressed genes relative to non-expressed genes. In contrast, for the HOT 500 m dataset, expressed genes were not enriched in core genes, which we speculate may be due to the activity of the microbial community at this depth (see Conclusions section below). Overall, however, the data support the broad trend that highly expressed genes are more likely to belong to an orthologous set shared across multiple taxa.

The differential representation of core genes within expressed and non-expressed genes may influence the relative sequence conservation levels of these two gene fractions. Gene acquisition from external sources (for example, homologous recombination, horizontal gene transfer (HGT)) is an important source of genetic variation in bacteria [29]. A conserved core genome is traditionally thought to undergo lower rates of recombination and HGT relative to more flexible genomic regions (for example, genomic islands) [30], though the horizontal transfer of core genes may also be common in some taxa [31]. A central limitation to shotgun sequencing datasets is that disparate sequences cannot be definitively linked to the same genome, making it challenging to evaluate the relative contributions of HGT, homologous recombination, and mutation to sequence divergence. Consequently, it is possible that the higher levels of sequence divergence observed in the non-expressed gene set are due in part to enhanced rates of HGT among the non-core genes that predominate in this gene set.

**Figure 5:**
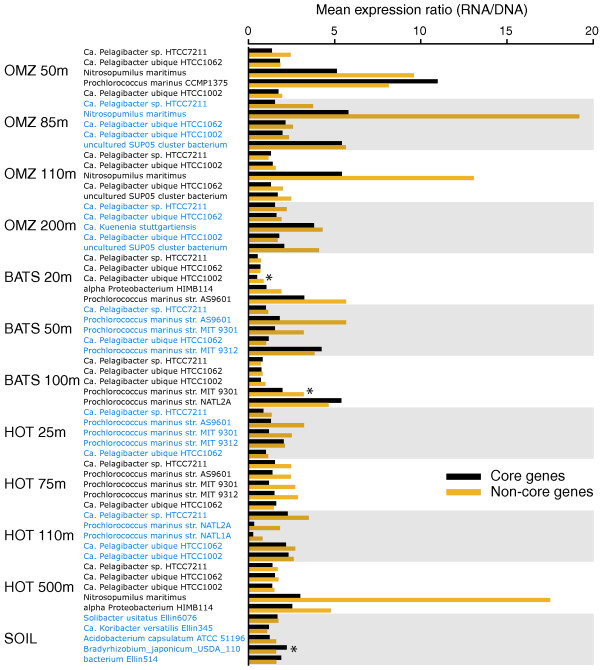
**Mean expression level of core and non-core genes across the five most abundant taxa per sample**. Per gene expression level is measured as a ratio - (Transcript abundance in RNA sample)/(Gene abundance in the DNA sample) - with abundance normalized to dataset size. 'Core' genes are determined individually for each taxon based on orthology with a closely related sister taxon, as described in the main text. Asterisks mark taxa for which expression ratios differed significantly between core and non-core genes (*P *< 0.001, *t*-test).

Surprisingly, within the expressed gene fraction, non-core genes were more highly expressed than core genes. Among the datasets representing the five most abundant taxa per sample (*n *= 60, as above), 80% showed higher expression levels (expression ratio) of non-core genes relative to core genes (Figure 5). Averaged across all of these taxa, the expression ratio was 34% higher in non-core genes relative to core genes (2.5 versus 1.9; *n *= 13,324 and 30,096, respectively; *P *< 0.00001). This pattern seemingly conflicts with studies based on cultured organisms. For example, a prior comparative survey of 17 bacterial proteomes showed a relative enrichment of peptides representing proteins encoded within the core genome [28]. Also, essential proteins necessary for organism survival have been shown to be expressed at higher abundances than nonessential proteins in cultures of both *Escherichia coli *[32] and *Pseudomonas aeruginosa *[33]. This observation indirectly links core genome representation and gene expression, as essential orthologs have been shown to be more broadly represented among diverse taxonomic groups than nonessential genes [34]. Our data, representing diverse taxa from the natural environment, raise the hypothesis that core genes are more likely to be expressed (above the level of detection at the sequencing depths used here). However, non-core genes, when expressed, are more likely to be expressed at higher levels. The high expression of non-core genes, also observed previously for *Prochlorococcus *[19], may reflect the importance of taxon-specific genes for adaptation to individual niches in a heterogenous environment [30].

### Functional patterns in expressed gene sets

The degree to which expressed gene sets share functional similarity across microbial communities from diverse habitats is unclear. Hewson *et al. *[16] observed shared functional gene content among metatranscriptome samples taken from the same depth zone (upper photic layer) at eight sites in the open ocean. Also, the four OMZ metatranscriptome datasets analyzed in this study have been shown to cluster separately from the corresponding metagenome datasets based on functional category abundances, suggesting similar expressed gene content across depths [35]. However, this clustering was likely influenced in part by variation in per-gene sequence abundance (evenness) between the metagenomes and metatranscriptome, and did not explicitly compare expressed and non-expressed gene fractions. Here, we explored functional differences between expressed and non-expressed genes (as defined above) within metagenome (DNA) samples, for which the relative read copy number per gene is more uniform than for metatranscriptome samples. To do so, the proportional abundance of KEGG gene categories and functional pathways was examined for five samples representing contrasting environments: the oxycline and lower photic zone of the coastal OMZ (50 m), the suboxic, mesopelagic core of the OMZ (200 m), the upper photic zone in the oligotrophic North Pacific (HOT 25 m), the deep, mesopelagic zone (HOT 500 m), and the soil from Harvard Forest.

**Figure 6:**
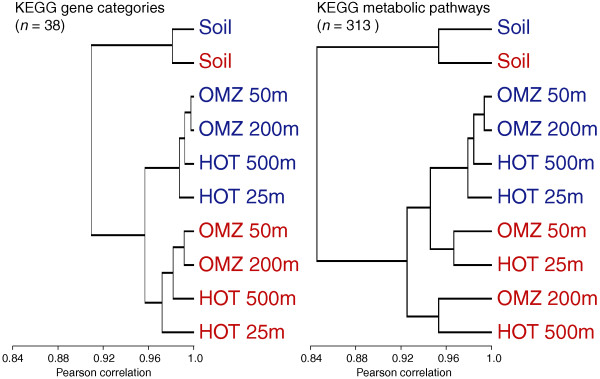
**Clustering of samples based on gene function**. Samples are hierarchically clustered based on the proportional abundance of KEGG gene categories and metabolic pathways in expressed (DNA+RNA; red) and non-expressed (DNA-only; blue) gene fractions in the DNA data from five representative samples.

**Figure 7:**
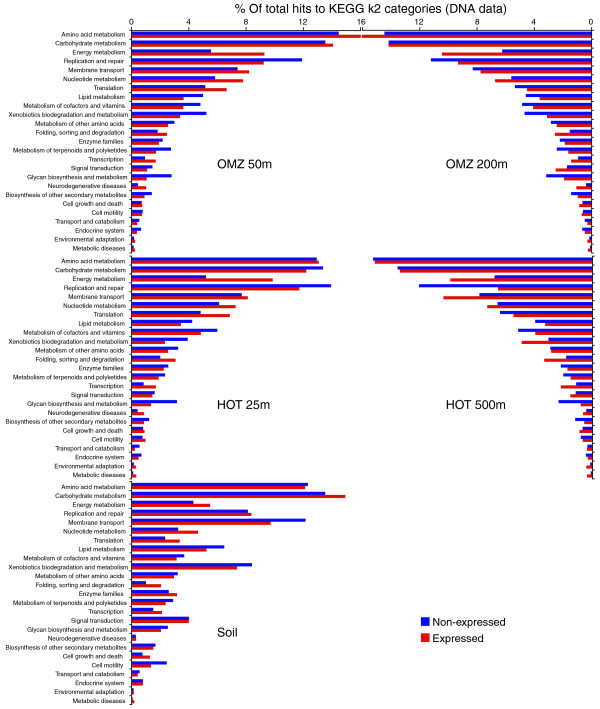
**Relative abundance of KEGG k2 functional categories (25 most abundant) across nr-reference genes identified in five representative DNA datasets**. Reference genes detected among the DNA reads were classified as unique to the DNA dataset (non-expressed) or shared between the DNA and RNA datasets (expressed).

**Figure 8:**
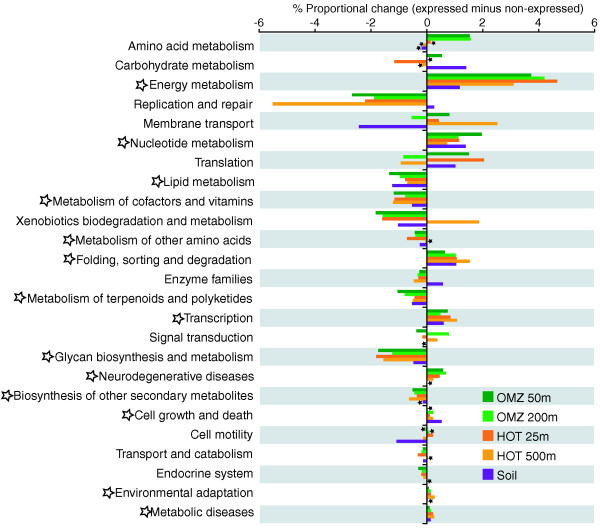
**Proportional change in KEGG category abundance between expressed and non-expressed gene fractions**. Data are shown for DNA datasets from five representative samples. Unfilled stars mark KEGG k2 categories in which the direction of change was consistent across all five samples. Small filled stars mark categories that did not differ significantly in relative abundance between expressed and non-expressed gene fractions (*P *> 0.05, chi-square). Only the 25 most abundant KEGG categories are shown.

**Figure 9:**
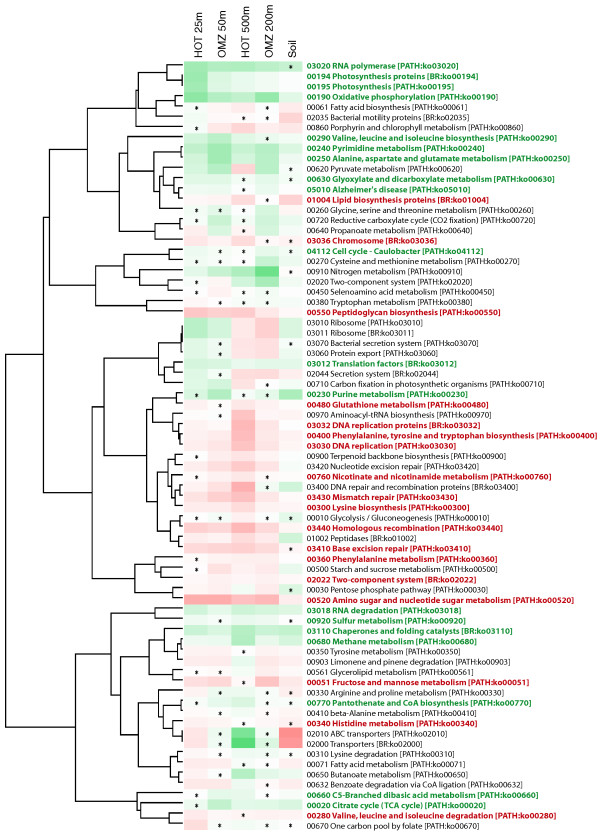
**Heat map showing the proportion increase (green) or decrease (red) of KEGG k3 functional pathway abundance between expressed and non-expressed gene fractions in DNA data from five representative samples**. Colored gene names mark KEGG pathways in which the direction of change was consistent across all five samples. All differences (abundance in expressed fraction versus abundance in non-expressed fraction) are significant (*P *< 0.0001, chi-square), unless marked with an asterisk. Only the 75 most abundant KEGG k3 pathways are shown. Genes are grouped by hierarchical clustering of Pearson correlation coefficients for each pairwise dataset comparison.

Hierarchical clustering based on correlations in gene category and functional pathway abundances indicated clear divisions among datasets. Not surprisingly, both the expressed and non-expressed fractions from the soil sample grouped apart from the ocean samples, highlighting functional differences between ocean and soil communities (Figures 6 and 7). Among the four ocean metagenomes, expressed gene sets clustered together to the exclusion of the non-expressed genes from the same samples (Figure 6). Indeed, shifts in functional gene usage between expressed and non-expressed fractions were broadly similar across all samples (Figures 8 and 9). Instances in which all five samples showed the same direction of change (increase or decrease) in KEGG gene category abundance occurred in 14 of the 25 functional categories shown in Figure 8 (marked by open stars), significantly higher (nine times) than random expectations if ignoring potential covariance between categories (*P *< 0.0002, chi-square). Notably, across all five samples, the expressed gene set was significantly enriched in genes involved in energy and nucleotide metabolism, transcription, and protein folding, sorting, and degradation (Figure 8). In contrast, the non-expressed gene set was enriched in genes mediating lipid metabolism and glycan biosynthesis and metabolism; in all ocean samples but not the soil sample, DNA replication and repair was also significantly overrepresented among non-expressed genes (*P *< 0.0004, chi-square). At the finer resolution provided at the KEGG pathway level, genes involved in oxidative phosphorylation, chaperones and protein folding catalysis, translation factors, and photosynthesis were consistently and significantly (*P *< 0.0001, chi-square) overrepresented among expressed genes in all samples, whereas genes of peptidoglycan biosynthesis, mismatch repair, and amino sugar and nucleotide sugar metabolism were proportionally more abundant in the non-expressed fraction (Figure 9). These data indicate broad similarities in functional gene expression across diverse microbial communities, with expressed gene pools biased towards tasks of energy metabolism and protein synthesis but relatively underrepresented by genes of cell growth (for example, lipid metabolism, DNA replication).

### Database-independent analysis

Our characterization of relative evolutionary rates in expressed versus non-expressed genes is based on sequence divergence relative to closest relatives in the sequence database (NCBI-nr). It is unclear to what extent this same trend may be detected within clusters of related sequences within our samples, independent of comparison to an external reference database. We therefore examined variability in amino acid divergence within clusters of expressed and non-expressed protein-coding sequences for five representative samples, including shallow and deep depths from the OMZ and HOT oceanic sites, and the surface soil sample (Table 6).

**Table 6:** Counts and mean percentage identity of amino acid sequence clusters for four representative samples

	**Cluster counts**^**a**^					**Mean percentage identity**^**d**^	
	
Sample	Total	Singleton	**DNA+RNA**^**b**^	**DNA only**^**c**^	**RNA only**^**c**^	DNA+RNA	DNA only
OMZ 50 m	213,683	180,311	1804	26,505	5063	77.0	85.2
OMZ 200 m	257,388	209,564	2712	40,401	4711	79.4	83.7
HOT 75 m	353,573	297,850	5681	44,163	5879	80.3	82.9
HOT 500 m	500,413	425,524	4677	66,151	4061	73.7	79.7
Soil	1,277,816	1,046,744	29,980	141,158	59,934	72.6	87.5

^a^CD-HIT clustering parameters: sequence identity 55% over local aligned region, with a length difference cutoff of 90%, and clustering to the most similar cluster (g = 1). ^b^Clusters containing both DNA- and RNA-derived sequences. ^c^Clusters containing only DNA- or RNA-derived sequences. ^d^Mean amino acid identity (relative to the cluster reference sequence) across all DNA sequences per cluster. BATS, Bermuda Atlantic Time Series; HOT, Hawaii Ocean Time Series; OMZ, oxygen minimum zone.

Mean identity per cluster was consistently higher for DNA sequences in non-expressed clusters compared to DNA sequences from expressed clusters (mean difference 5.3%; Table 6). This pattern is opposite to that observed in comparisons of sequences to external reference databases (above). However, we argue that this inverse pattern is indeed consistent with our hypothesis that expressed genes are more likely to be part of a core set shared across taxa (Figure 4). If this hypothesis is true, then the DNA-only cluster set (non-expressed genes) will be relatively enriched in non-core genes, including those present in only one taxon/genome and lacking any known homologs (for example, orphans) [36,37]. In environmental sequence sets, if these sequences appear multiple times, they are more likely to be identical, or nearly so, because they come from a single taxon population and therefore cluster only with themselves (homologs from other taxa are by definition absent and will not fall into the cluster).

**Figure 10:**
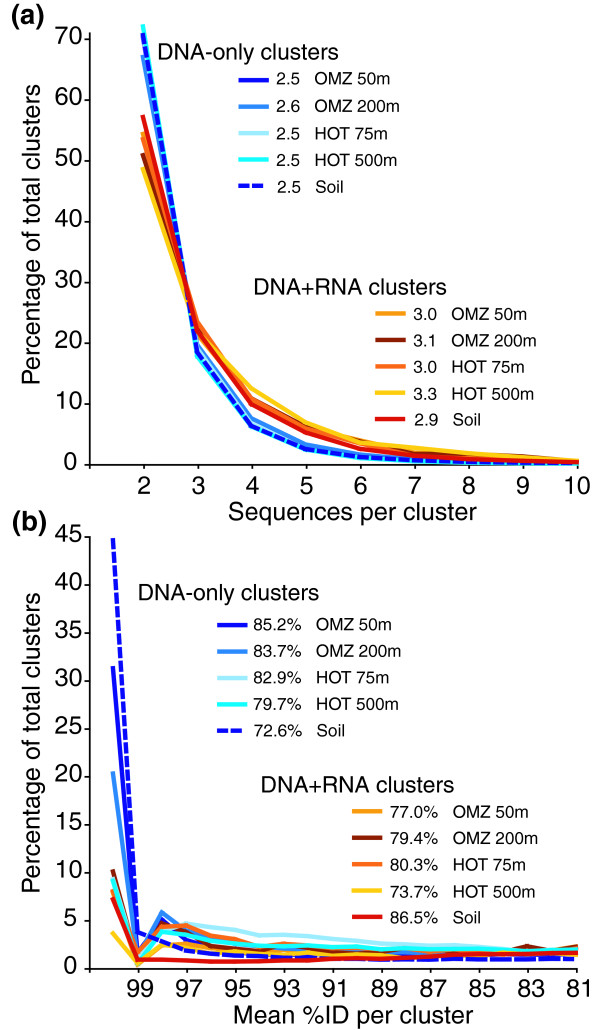
**Database-independent cluster statistics**. **(a) **Size and **(b) **percentage identity of clusters containing amino acid sequences present only in DNA datasets or in both DNA + RNA datasets from five representative samples. Cluster sizes are based on counts of only the DNA-derived sequences within each cluster type. Numbers in legends indicate mean cluster size (a) and mean amino acid identity (b). Amino acid sequences were clustered above a threshold identity of 55%.

In contrast, if expressed genes are more likely to fall within the core genome, clusters containing both DNA- and RNA-derived sequences (that is, expressed sequences) will be relatively enriched in homologs that occur across multiple divergent taxa. By definition, therefore, DNA+RNA clusters will be relatively enriched in sequences differing at both the population level and at higher taxonomic levels (for example, 'species'), while DNA-only clusters will be enriched in sequences differing only at the population level. Given this explanation, we would predict that DNA+RNA clusters (with RNA sequences excluded) are larger than DNA-only clusters and that the DNA-only cluster set as a whole is enriched in high identity clusters. Indeed, DNA+RNA clusters are, on average, approximately 20 to 33% larger than DNA-only clusters (RNA sequences not included in counts) and DNA-only cluster sets, notably those of the OMZ samples, are enriched in clusters with identities greater than 98% (Figure 10). These data indicate that expressed gene clusters recruit a larger and more diverse set of sequences, consistent with the hypothesis that expressed genes are more likely to represent core genes shared across taxa. More generally, the contrast between this self-clustering approach and the BLAST-based comparisons (above) demonstrates how divergence measurements taken relative to an external top match reference can differ from those relative to a top match internal reference from the same dataset, with the latter more likely to involve comparisons between highly related sequences from the same strains/populations.

### GC content and amino acid usage differ between expressed and non-expressed genes

The discrepancy in sequence conservation between expressed and non-expressed genes coincided with differences in nucleotide composition and amino acid usage between these two sequence pools. GC content was substantially higher in the soil compared to the ocean samples (approximately 20 to 25% enrichment) and consistent between the DNA and RNA pools (Table 7). In contrast, across all 11 ocean samples, RNA-derived protein-coding sequences were significantly elevated in GC relative to those from the DNA (mean RNA-DNA difference, 6%; Table 7), suggesting a broad shift towards GC enrichment in the expressed gene pool. Surprisingly, however, DNA sequences corresponding to expressed genes consistently had a lower GC content than DNA reads matching non-expressed genes (mean difference, 1.9%). These data suggest that the DNA versus-RNA discrepancy in GC content may be driven by a subset of transcripts in the RNA pool, likely those at high abundance. Indeed, analysis of the RNA reads from one sample (OMZ 50 m) showed a progressive increase in GC content with transcript abundance (when transcripts are subdivided into four categories (top 10%, 1%, 0.1% 0.01%) based on the rank abundance of the genes they encode (data not shown).

**Table 7:** GC percentages (averaged over all reads) in open reading frames identified using Metagene

		DNA reads			RNA reads
			
Site	Depth (m)	**All**^**a**^	**DNA only**^**b**^	**DNA+RNA**^**c**^	**All**^**a**^
OMZ	50	37.6	38.2	36.0	42.8
	85	38.2	38.4	36.7	44.9
	110	41.1	42.6	38.1	42.8
	200	40.9	41.9	38.6	45.6
BATS 216	20	34.5	35.4	33.8	42.8
	50	35.2	36.4	33.9	40.3
	100	33.7	34.7	32.9	37.8
HOT 186	25	35.5	36.0	34.8	44.0
	75	34.9	35.2	34.4	41.4
	110	36.0	36.4	35.0	40.0
	500	43.2	43.1	43.5	50.6
Soil	Surface	62.7	63.1	62.5	62.6

^a^All reads identified as 'protein-coding' via significant BLASTX matches to NCBI-nr (bit-score > 50), with GC content then estimated for Metagene-called open reading frames within this read set. ^b^Reads matching genes detected only in the DNA data. ^c^Reads matching genes detected in both the DNA and RNA data. BATS, Bermuda Atlantic Time Series; BLAST, Basic Local Alignment Search Tool; GC, guanine-cytosine; HOT, Hawaii Ocean Time Series; HSP, high-scoring segment pair; NCBI-nr, National Center for Biotechnology Information non-redundant protein database; OMZ, oxygen minimum zone.

**Figure 11:**
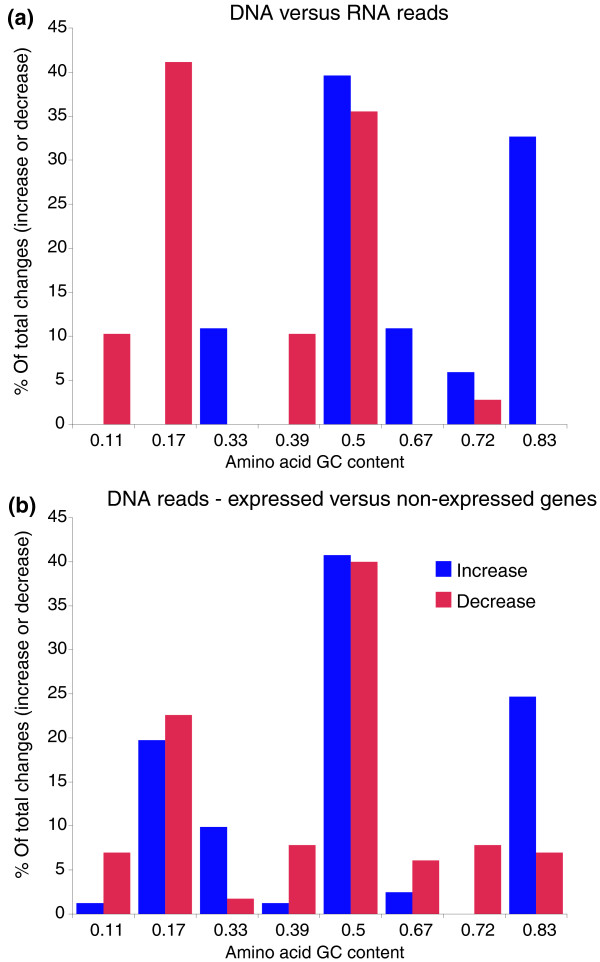
**Amino acid usage changes based on GC content of synonymous codons in ocean communities**. **(a,b) **Charts include only those amino acids whose frequency significantly increased (blue) or decreased (red) in the RNA relative to the corresponding DNA reads (a) or in the expressed genes relative to the non-expressed genes in the DNA reads only (b). Significant increases/decreases are categorized based on the GC content (x-axis) of the amino acid, where GC content = (Number of GC bases among synonymous codons)/(Number of total bases among synonymous codons). Data from all 11 DNA/RNA ocean samples are pooled; these charts exclude the soil data, in which GC enrichment in the RNA was not observed.

**Figure 12:**
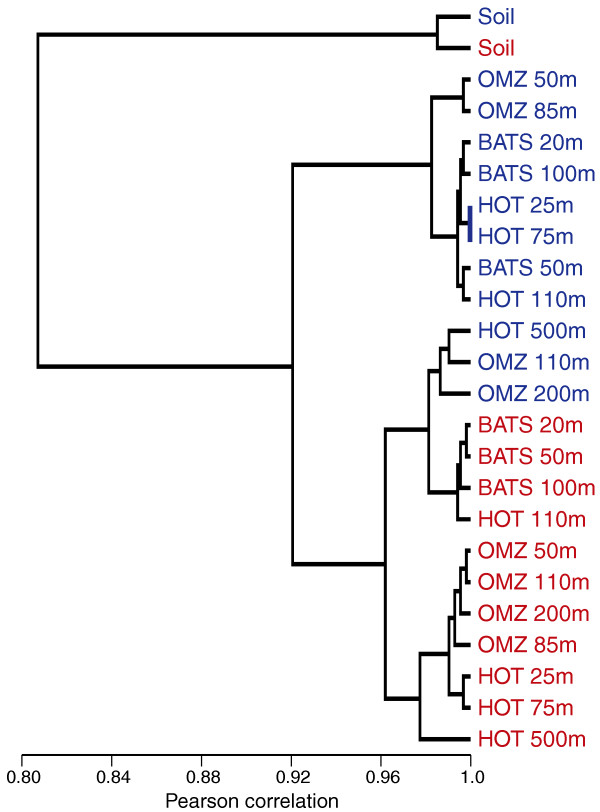
**Relatedness of DNA (blue) and RNA (red) datasets as determined by amino acid proportions**. Dendrograms are based on hierarchical clustering of Pearson correlation coefficients for each pairwise comparison of amino acid proportions.

**Figure 13:**
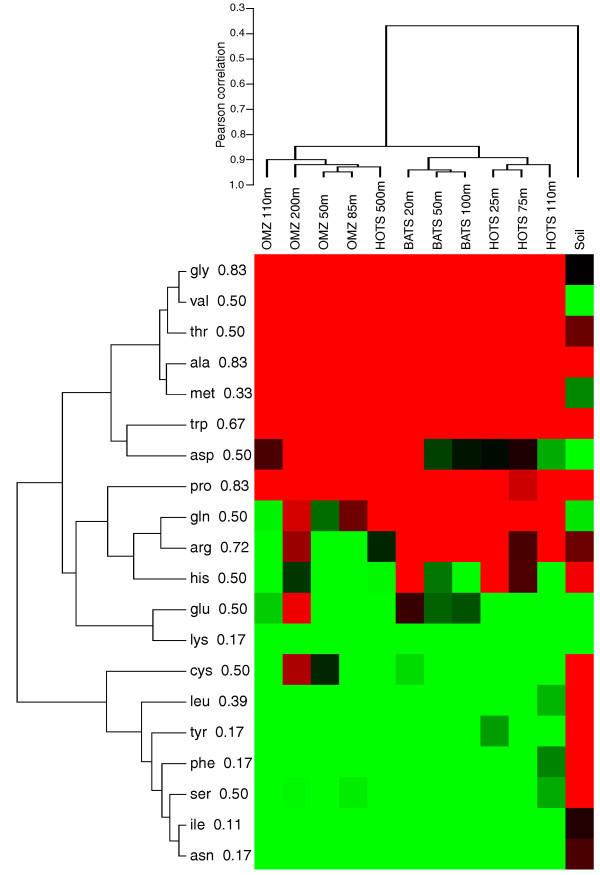
**Clustering of sample datasets based on proportional amino acid change**. Heat maps show the relative magnitudes of the proportional change (from DNA to RNA) in each sample set. Red = increase, green = decrease, black = zero. Dendrograms are based on hierarchical clustering of Pearson correlation coefficients for each pairwise dataset comparison. Numbers beside amino acid names indicate GC content, calculated as: (Number of GC bases among synonymous codons)/(Number of total bases among synonymous codons).

**Figure 14:**
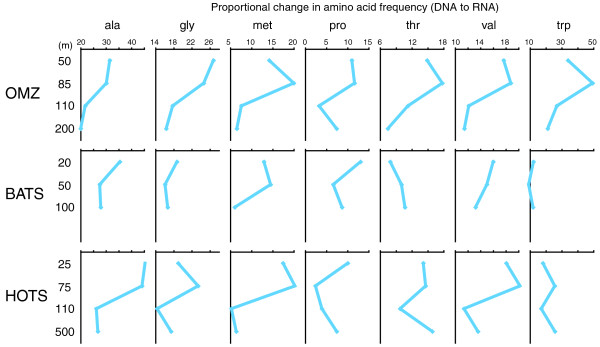
**Proportional changes in amino acid usage decrease with depth**. Data include the seven amino acids with the greatest proportional increase from DNA to RNA. Proportional change = ((Amino acid proportion in RNA) - (Amino acid proportion in DNA))/(Amino acid proportion in DNA) × 100.

Consistent with the GC pattern, amino acid usage of protein-coding sequences differed significantly between the DNA and RNA samples (Table 8, Figures 11, 12, 13, and 14). Notably, with the exception of three ocean samples (HOT 500 m, OMZ 110 m and 200 m) and the outlying soil sample, RNA datasets from diverse regions and depths grouped separately from DNA samples when clustered based on amino acid frequencies (Figure 12), suggesting a global distinction between the metagenomic and metatranscriptomic amino acid sequence pools in marine microbial communities. Indeed, of 240 comparisons of amino acid proportions in DNA versus RNA datasets (12 DNA/RNA samples × 20 amino acids), 227 (95%) involved a significant change in amino acid frequency, with 114 involving an increase and 113 involving a decrease in frequency from DNA to RNA (*P *< 0.0002, chi-square; Table 8, Figure 13). (The high proportion of significant changes is due to the large sample sizes in the analysis.) On average, alanine, glycine, and tryptophan (high GC content) underwent the largest proportional increases from DNA to RNA, while lysine, isoleucine, and asparagine (low GC content) all decreased substantially in frequency. These shifts were largely consistent among ocean samples, but clearly distinct from the pattern observed in soil, where several amino acids changed in frequency in the direction opposite to that in the ocean samples.

**Table 8:** Proportional change^a ^in amino acid usage in RNA datasets compared to DNA datasets

Amino acid		OMZ				BATS 216			HOTS 186				
			
	**GC**^**b**^	50 m	85 m	110 m	200 m	20 m	50 m	100 m	25 m	75 m	110 m	500 m	Soil
Ala	0.83	31.5	30.2	21.8	20.1	35.5	27.6	28.0	45.4	44.2	26.2	26.8	5.5
Gly	0.83	26.8	24.7	17.8	16.4	18.7	16.1	16.7	19.0	23.4	14.4	-10.2	0.0
Pro	0.83	11.0	11.7	3.3	7.4	13.1	6.6	8.7	10.1	2.4	4.0	6.8	5.1
Arg	0.72	-3.3	-3.2	-11.7	1.8	23.2	14.2	9.7	11.9	0.9	4.1	-6.7	1.3
Trp	0.67	34.0	49.5	27.0	21.2	12.2	9.2	11.9	18.2	25.7	17.1	-9.3	16.3
Cys	0.50	-0.5	-5.1	-6.9	2.1	-2.6	-6.6	-9.5	-10.3	-8.9	-11.8	17.5	3.9
Asp	0.50	3.3	5.6	0.9	8.0	3.0	-0.8	-0.2	-0.1	0.4	-2.0	-2.9	-3.6
Glu	0.50	-7.0	-11.1	-2.4	2.8	0.7	-1.2	-1.0	-6.6	-8.9	-5.6	-21.7	-9.0
His	0.50	-4.1	-5.4	-7.8	-0.6	4.1	-1.4	-3.4	9.7	0.9	-4.5	-28.3	2.9
Gln	0.50	-1.3	1.3	-2.9	2.5	9.3	6.4	6.8	9.3	3.8	4.2	-10.8	-2.7
Ser	0.50	-5.4	-2.8	-3.4	-2.9	-8.5	-7.4	-5.2	-7.6	-6.3	-2.0	6.4	4.1
Thr	0.50	14.9	17.8	11.3	7.5	7.9	10.0	10.7	14.2	14.6	9.8	-17.4	1.3
Val	0.50	17.7	18.8	12.2	11.5	16.0	15.0	13.2	18.1	20.2	11.4	7.5	-4.1
Leu	0.39	-9.7	-11.0	-10.4	-8.1	-6.6	-6.7	-5.3	-7.2	-6.9	-2.2	7.8	4.4
Met	0.33	14.2	20.0	7.6	6.6	13.0	14.5	6.1	17.5	20.3	5.3	-0.5	-1.6
Phe	0.17	-10.5	-9.1	-6.9	-10.2	-15.9	-11.3	-9.6	-8.4	-5.8	-1.6	-3.6	4.3
Lys	0.17	-22.3	-28.6	-9.5	-17.8	-13.7	-8.4	-6.6	-25.1	-23.4	-11.5	15.9	-26.3
Asn	0.17	-23.6	-20.5	-14.8	-18.0	-25.1	-20.2	-19.2	-24.9	-23.0	-17.4	13.6	0.9
Tyr	0.17	-8.4	-6.7	-5.3	-4.8	-15.2	-9.3	-11.0	-1.9	-5.6	-7.7	25.7	5.4
Ile	0.11	-19.3	-21.8	-11.8	-18.2	-22.5	-18.2	-18.5	-23.0	-20.6	-17.5	-3.7	0.4

^a^((Proportion in RNA) - (Proportion in DNA))/(Proportion in DNA) × 100. ^b^GC content = (Number of guanine or cytosine bases among synonymous codons)/(Number of total bases among synonymous codons). BATS, Bermuda Atlantic Time Series; HOT, Hawaii Ocean Time Series; OMZ, oxygen minimum zone.

Among the ocean datasets, DNA-RNA shifts in amino acid frequency were strongly related to amino acid GC content (Figure 11a; see Materials and methods). Amino acids with an intermediate GC content (0.5) constituted equivalent fractions, 40% and 36%, of the total number of amino acid frequency increases and decreases, respectively. Strikingly, amino acids with GC content below 0.5 were significantly less abundant in the RNA, being involved in 61% of all decreasing DNA-RNA amino acid frequency changes. In contrast, frequency increases were dominated by amino acids enriched in GC: 50% of increases involved amino acids with GC greater than 0.5, significantly higher than the representation of these amino acids in changes involving a decrease (3%). A similar, but less dramatic, shift in amino acid usage is observed when the DNA reads were binned into expressed and non-expressed gene sets (Figure 11b). In general, the magnitude of the proportional shift in amino acid usage decreases with depth in the water column (Figure 14). This pattern may reflect an overall decrease in microbial activity with depth, such that the transcriptome, less weighted by highly expressed and highly conserved genes, more closely resembles the metagenome as activity declines. Together, these results suggest a significant shift towards GC-rich amino acids in the expressed gene pool.

Prior studies describe a relationship among gene expression level, sequence conservation, and amino acid usage [38-42]. Specifically, significant enrichment in GC-rich amino acids among highly expressed genes has been demonstrated for individual bacterial taxa, including *Prochlorococcus *[38,39]. GC richness in expressed genes is potentially driven by a combination of factors, including selection against metabolically costly amino acids (for example, AT-enriched phenylalanine and tyrosine) [40], or selection against AT-richness in highly expressed genes. Alternatively, this pattern may stem from an overall enhanced conservation level in highly expressed genes [12]. Assuming an underlying GC-to-AT mutational bias, which may be a universal trend in bacteria [41,42], selectively constrained genes are predicted to retain a GC-rich signature relative to less-constrained genes. Therefore, the proportional increase in GC-enriched amino acids in expressed genes compared to non-expressed genes in this study is consistent with our observation of enhanced sequence conservation in the expressed community gene pool, and confirms a fundamental distinction in amino acid usage related to gene expression level.

## Conclusions

Microbial metagenomes and metatranscriptomes are amalgams of thousands of taxonomically and functionally diverse microorganisms, each of which experiences unique evolutionary pressures. Such complexity might be expected to preclude the detection of bulk evolutionary signals in meta-omic data. Here, we show broad trends in protein coding sequence conservation that transcend variation in both taxonomic composition and habitat type.

Specifically, we confirm that the hypothesized positive relationship between gene expression level and sequence conservation, which has been well established for individual taxa under experimental conditions [11], is a universal trend across diverse microbial communities in both marine and terrestrial environments. Detecting this trend required binning genes into broad categories (expressed versus non-expressed) based on the detection of transcripts, which depended, in part, on the depth of sequencing per sample. Deeper sequencing would reveal a greater proportion of the expressed gene pool and potentially lead to more accurate measurements of expression level for low frequency genes [22,23]. However, the tremendous taxonomic diversity inherent in microbial communities, as well as the temporal heterogeneity of the environment in which these communities exist, likely confounds any attempt to predict protein conservation based on transcript abundance on a gene-by-gene basis using meta-omic data. Nonetheless, the broad discrepancy in sequence conservation between expressed and non-expressed gene fractions is significant, operates consistently across diverse taxa (Figure 4), and confirms that expression level is a primary determinant of evolutionary rate in naturally occurring microorganisms.

The mechanism linking evolutionary rates and expression level is still debated. Sequence conservation in highly expressed proteins has been hypothesized to be driven by selection acting to minimize the costs of protein misfolding, which should increase in tandem with expression level (protein copy number per cell) [14], though the harmful effects of misfolding have been brought into question [43]. This selection for 'translational robustness/accuracy' is predicted to be largely decoupled from a protein's functional importance [13,14,24]. Here, using mRNA abundance as a proxy for expression level, our results demonstrate broad commonalities in expressed gene content across communities in widely different habitats (ocean versus soil). These data indicate a trend toward genes of protein synthesis and energy metabolism in the more actively expressed gene fraction and toward genes of cell replication and growth in the less expressed fraction. Additionally, this finding, in the context of our results demonstrating enhanced sequence conservation among expressed genes, indirectly suggests that the expression-conservation relationship may partially be constrained by protein function. However, these data cannot be used to justify this conclusion, since both gene expression level and functional importance may independently co-vary with protein evolution rates, as has been demonstrated for isolates of *Pseudomonas aeruginosa *[33]. Though characterizing the mechanism linking gene expression level and evolutionary rate is beyond the scope of this study, metatranscriptomic data may inform future studies exploring the relative effect of protein function on sequence conservation.

We show that the expressed gene set, compared to the non-expressed set, is more likely to contain genes that belong to an orthologous core genome shared across closely related sister taxa. This pattern was broadly consistent across the marine samples and the soil sample. Interestingly, the overrepresentation of expressed genes within the core set was not observed in the HOT 500 m sample (Figure 4), which we hypothesize may be related to an overall decline of metabolic activity at deeper depths within the water column. Microbial transcriptomes can vary significantly in response to the growth phase of the organism [44-46]. In less actively growing communities (for example, stationary phase), expression level might be more uniform across the genome (that is, background expression), with both core and non-core genes having a relatively equal probability of detection. In contrast, in actively growing communities, the distribution of transcripts might become dominated by a subset of highly expressed genes (for example, genes mediating energy metabolism, membrane transport), as we have observed in other samples. If such genes fall within the core genome, core genome representation in the expressed gene set would be predicted to be greater in more active communities.

Our datasets highlight similarities in gene expression and sequence evolution across very different microbial habitats, but differ markedly in other attributes. Notably, the soil community was a clear outlier with respect to functional gene content (Figures 6, 7, and 9) and amino acid usage (Figures 12 and 13), likely due to the distinct community composition of this habitat (Figure 4). However, given our analysis of a single soil metatranscriptome, and the use of different RNA extraction kits for soil versus marine samples (see Materials and methods), we urge caution when comparing microbial community composition between soil and marine datasets. A more comprehensive comparison of taxonomy and functional gene expression would involve extended metatranscriptome sampling across multiple soil types (and locations), as well as optimization of RNA extraction protocols to ensure unbiased lysis of all microorganisms. Such an analysis was not the focus of this study. However, the inclusion of the soil sample confirmed a positive relationship between expression level and sequence conservation at both the genome and community levels (Figures 3 and 4), as well as an overrepresentation of core genes within the highly expressed gene set (Figure 4). Though it is possible that such patterns may not be observed in other sample types, or following different extraction protocols, our results provide strong evidence for universal features of protein-coding gene evolution in natural microbial communities.

The composition of metatranscriptomic and metagenomic sequence datasets depends not only on intrinsic biological factors (for example, community composition, metabolic state) but also on the physical and chemical environment at the time of sampling. Furthermore, interpretation of the resulting data can vary based on the analytical method (for example, database-dependent versus -independent analyses, as shown here) and on the availability and biases of the reference sequences to which the data are compared. Here, we attempt to rule out potential database artifacts by analyses at both the community and genome level. In so doing, our results suggest that environmental meta-omic datasets, despite their inherent complexity, can inform theoretical evolutionary predictions and reveal universal trends across ecologically and phylogenetically diverse microbial communities.

## Materials and methods

We examined protein-coding sequences in coupled microbial metagenomes and metatranscriptomes from multiple depths at three distinct oceanographic sites and from surface soil in a temperate forest (Table 1). Ocean datasets (excluding the HOT 110 m RNA sample, which was sequenced in this study) were generated in prior studies using the Roche 454 Genome Sequencer with FLX series chemistry and extracted from public databases (see Table 1 for accession numbers). Sequences from the soil sample were generated in this study (detailed below) and are available in the NCBI Sequence Read Archive under accession [SRA028811].

### Soil sample collection and DNA/RNA isolation

Soil was collected from within a transition hardwood-white pine/hemlock forest in the Prospect Hill Tract of Harvard Forest (Massachusetts, USA; 42.54 N 72.18 W; elevation, 385 m) on 27 September 2010. Two cores were taken from 1 to 10 cm below the leaf horizon using a 10 mm diameter soil corer. These cores were homogenized, placed into 50 ml Falcon tubes, flash frozen in liquid nitrogen, and transported to the lab on dry ice. Visible pieces of plant material were removed from soil subsamples with sterile forceps. During plant removal, the subsamples were placed on a sterile surface, laid within a bed of dry ice. Total microbial DNA and RNA were then extracted from these subsamples using PowerSoil Total RNA and DNA isolation kits (MoBio, Carlsbad, CA, USA), according to the manufacturer's protocol. This extraction protocol differs from the method used to generate the marine datasets, which employed the *mir*Vana™ miRNA Isolation kit (Ambion, Austin, TX, USA) for RNA isolation [17,19,23,35]. It is possible that these kits may lyse different microbial taxa at varying efficiencies. As this possibility has not been assessed, we urge caution when comparing the compositions of soil and marine communities based on metatranscriptome data, though this was not the focus of our study. Total DNA was quantified and used directly for pyrosequencing. Total RNA was further processed, as described below.

### rRNA subtraction, RNA amplification and cDNA synthesis

Total RNA was amplified and prepared for pyrosequencing using established protocols. Briefly, the proportion of ribosomal RNA transcripts (bacterial and archaeal 16S and 23S rRNA and eukaryotic 18S and 28S rRNA) in total soil RNA was reduced via a published subtractive hybridization protocol using sample-specific rRNA probes [23]. Following rRNA subtraction, total RNA was amplified as described previously using a modification of the MessageAmp™ II-Bacteria kit (Ambion) [17,19]. Briefly, total RNA was polyadenylated and converted to double-stranded cDNA via reverse transcription. cDNA was then transcribed *in vitro *(37°C, 12 to 14 h) to produce microgram quantities of single-stranded antisense RNA. The amplified products (approximately 5 to 10 μg aliquot) were converted back to double-stranded cDNA using the SuperScript^® ^III First-Strand Synthesis System (Invitrogen, Carlsbad, CA, USA) for first-strand synthesis (random hexamer priming), and the SuperScript™ Double-Stranded cDNA synthesis kit (Invitrogen) for second-strand synthesis. cDNA was then purified (QIAquick PCR purification kit, Qiagen, Valencia, CA, USA), digested with BpmI (37°C, 3 h) to remove poly(A) tails, and used directly for pyrosequencing.

### Pyrosequencing

Soil DNA and cDNA were purified for sequencing via the Agencourt^® ^AMPure^® ^kit (Beckman Coulter Genomics, Danvers, MA, USA) and used for the generation of single-stranded DNA libraries and emulsion PCR according to established protocols (454 Life Sciences, Roche, Branford, CT, USA). Clonally amplified library fragments were sequenced with full plate runs on a Roche Genome Sequencer FLX instrument using Titanium chemistry. Pyrosequencing datasets generated during this study have been deposited in the NCBI Sequence Read Archive under accession numbers listed in Table 1.

### Data analysis

#### Homology searches

Pyrosequencing reads matching ribosomal RNA genes were identified in cDNA and DNA datasets by BLASTN searches against a custom database of prokaryotic and eukaryotic small and large subunit rRNA sequences (5S, 16S, 18S, 23S and 28S rRNA) taken from microbial genomes and the ARB SILVA LSU and SSU databases [47]. Reads matching rRNA with bit scores >50 were identified and removed from further analysis. Replicate sequences sharing 100% nucleotide similarity and length, which may represent artifacts generated by the pyrosequencing protocol [23,48], were identified among non-rRNA sequences using the open-source program CD-HIT [49] and removed from each dataset. Non-replicate, non-rRNA sequences were characterized by BLASTX searches against NCBI-nr, KEGG, and assembled protein sequences taken from the GOS database. Protein-coding sequences were identified as the top reference (database) gene(s) matching each read above a bit score of 50. When reads matched multiple genes with equal bit score, each matching gene was considered a top hit, with its representation scaled proportionate to the number of genes sharing the same bit score.

#### Amino acid identities, relative taxon abundance, and expression ratio

Local amino acid identities were recovered from within the top HSP for each sequence having a significant match (bit score >50) to a reference gene in the nr database; gaps were not included in identity calculations. Identities of multiple reads matching the same reference gene were averaged to obtain per gene identities, and these values were then averaged across all reference genes per dataset. Relative taxon abundance per sample was determined as the number of sequence reads matching the same reference taxon as a top hit in BLASTX searches of NCBI-nr. The relative transcriptional activity (expression level) per expressed gene was normalized to account for variations in gene abundance in the DNA pool, as calculated by the expression ratio: (RNA reads per gene/Total RNA reads matching NCBI-nr)/(DNA reads per gene/Total DNA reads matching NCBI-nr)

#### Core genome

The proportional representation of expressed and non-expressed genes in the orthologous gene set shared between taxa was determined separately for the top five most abundant organisms in each of the samples. The core genome of *Prochlorococcus *sp., which was common in our samples, was defined as those genes present across all 12 of the sequenced *Prochlorococcus *strains, based on the analysis of [50]. For all other taxa, a core gene set was more broadly defined by reciprocal BLASTP searches against a closely related sister taxon (bit score cutoff = 50). Sister taxa used for core genome identification, and the number of genes shared between taxa, are listed in Table 5.

#### Hierarchical clustering

Average-linkage clustering was performed in Cluster 3.0 using Pearson coefficients (centered) derived from pairwise correlations of KEGG functional category abundances (Figures 6 and 9), amino acid proportions (Figure 12), or proportional changes in amino acid usage (Figure 13). KEGG category clustering was based on expressed and non-expressed gene sets within the DNA data only, as defined in the main text based on matches to nr reference proteins. Abundance per KEGG category was calculated as the total number of reads per category as a proportion of total reads matching the KEGG hierarchy at either the category (Figure 8) or pathway level (Figure 9).

#### Database-independent cluster analysis

Sequence divergence was determined for clusters of related sequences within pooled DNA + RNA datasets. To avoid spurious clustering of non-coding regions (for example, small RNAs (sRNAs)), amino acid sequences present in the DNA and RNA reads (those with significant matches to the nr database) were identified using the *ab initio *gene-finding program Metagene [51]. (Comparisons of amino acid sequences determined from Metagene and sequences determined from BLAST HSP regions produced nearly identical results.) Sequences derived from DNA and RNA reads were pooled and clustered using the program CD-HIT [49] applying a low sequence identity threshold (55%) over the aligned region. Resulting clusters contained RNA-derived sequences only, DNA-derived sequences only, or both DNA- and RNA-derived sequences. For all clusters containing DNA-derived sequences only (non-expressed) or DNA + RNA-derived (expressed) sequences, the divergence between each DNA-derived sequence and the reference (longest) sequence per cluster was calculated along the length of the aligned region. These values were averaged to estimate divergence per cluster. Clusters containing only one sequence (singletons) and clusters in which the reference sequence was the only DNA sequence were excluded from further analysis. For clusters in which the reference sequence was RNA-derived, but that contained only one DNA sequence, the percentage identity of the sole DNA sequence was recorded. Divergence per cluster was then averaged for all expressed and non-expressed sequence/gene clusters.

#### Amino acid usage

We compared amino acid usage between DNA and RNA datasets and DNA datasets parsed into expressed versus non-expressed gene categories as above. Specifically, for each dataset, reads matching nr proteins (see above for BLASTX descriptions) were examined to assess amino acid usage and GC content, using two approaches. First, amino acids were counted across all HSPs in alignments between sequence reads and their closest match in the nr database. Second, amino acids were counted across open reading frames that were identified using Metagene [51]. These two methods returned nearly identical results; we therefore restrict our discussion to the Metagene data. Amino acids were then categorized based on GC content, where GC content was measured by the relative proportion of Gs and Cs among the synonymous codons for each amino acid (for example, alanine has four synonymous codons; 10 of the 12 total bases (0.83) representing these codons are either a G or C).

Proportional changes in amino acid usage, KEGG gene category and pathway abundances, and core genome representation in expressed and non-expressed gene fractions were compared using chi-square tests. Mean amino acid identities and expression ratios were compared using standard two-tailed *t*-tests. Significance was determined at alpha <0.05 following Bonferroni corrections for multiple comparisons.

## Abbreviations

BATS: Bermuda Atlantic Time Series; GOS: Global Ocean Sampling; HGT: horizontal gene transfer; HOT: Hawaii Ocean Time Series; HSP: high-scoring segment pair; KEGG: Kyoto Encyclopedia of Genes and Genomes; NCBI-nr: National Center for Biotechnology Information non-redundant protein database; OMZ: oxygen minimum zone.

## Competing interests

The authors declare that they have no competing interests.

## Authors' contributions

EFD and FJS conceived of the study. FJS carried out the computational analyses of pyrosequencing data and drafted the manuscript. JME participated in the computational sequence and statistical analysis, provided important coding support, and helped revise the manuscript. JAB helped design the study, provided critical analytical and data acquisition support, and helped revise the manuscript. AKS participated in data acquisition and computational sequence analysis and helped revise the manuscript. EFD coordinated the overall goals of the study, directed the analytical work, and helped to draft the manuscript. All authors read and approved the final manuscript.
